# PD-1 inhibitor improves radiosensitivity by tumor vessel normalization

**DOI:** 10.1038/s41416-025-03315-8

**Published:** 2025-12-26

**Authors:** Shengnan Hao, Dashan Ai, Quanxin Wang, Xiangyan Zhang, Xiangli Ma, Ihsuan Tseng, Jingyi Shen, Yun Chen, Qi Liu, Jiaying Deng, Hongcheng Zhu, Zhaolu Kong, Kuaile Zhao

**Affiliations:** 1 Department of Radiation Oncology, Fudan University Shanghai Cancer Center, Shanghai, China; 2 Department of Oncology, Shanghai Medical College, Fudan University, Shanghai, China; 3 Shanghai Key Laboratory of Radiation Oncology, Shanghai, China; 4 Department of Urology, Fudan University Shanghai Cancer Center, Shanghai, China; 5 The Institute of Radiation Medicine, Fudan University, Shanghai, China; 6 Department of Radiation Oncology, Shanghai Proton and Heavy Ion Center, Shanghai, China

**Keywords:** Radiotherapy, Radiotherapy

## Abstract

**Background:**

Host immunity status and hypoxia are the hallmarks of radiosensitivity. Induction of anti-PD-1 immunotherapy demonstrates promise in locally advanced tumor radiotherapy, but whether anti-PD-1 immunotherapy improves radiosensitivity is unclear.

**Methods:**

In vivo experiments were performed in mouse models (4T1 and LLC) treated with anti-PD-1 antibodies or X-ray irradiation. Tumor tissues were subjected to bulk-RNA sequencing. The immune cell profile was characterised by flow cytometry. The hypoxia level was detected by immunofluorescence and Hypoxyprobe and measured using the hypoxia gene score. Vessel normalization was determined by the pericyte–endothelial cell ratio. Anti-CD4 and anti-CD8 monoclonal antibodies were used to deplete CD4^+^ and CD8^+^ immune cells, respectively, in mice. The differences among anti-PD-1 immunotherapy, anti-PD-1 immunotherapy without CD4^+^ cells, and anti-PD-1 immunotherapy without CD8^+^ cells were compared using transcriptome analysis. The spatial immunophenotype was investigated using multi-marker immunofluorescence and HALO analyses.

**Results:**

Induction immunotherapy increased radiosensitivity and maximized anti-tumor response compared with concurrent administration of immunotherapy by mitigating hypoxia. Immune cell profile analysis showed that the number of CD4^+^ IFNγ^+^ T cells, but not CD8^+^ IFNγ^+^ T cells, increased significantly after the induction of anti-PD-1 therapy combined with radiotherapy compared with concurrent radioimmunotherapy. Using antibodies to deplete CD4^+^ or CD8^+^ T cells, we confirmed that CD4^+^ T cells contribute to PD-1 inhibitor-induced vessel normalization and reduced hypoxia. Spatially, more CD4^+^ T cells infiltrate the tumor invasive margin and are located around CD31^+^ endothelial cells after anti-PD-1 immunotherapy.

**Conclusions:**

PD-1 inhibitors improve radiosensitivity through vasculature and immune reprogramming, and vessel normalization may be a biomarker for distinguishing patients who will benefit from radiotherapy after induction immunotherapy.

## Background

Approximately 60% of all cancer patients receive radiotherapy during the course of their illness to improve local disease control and quality of life [1]. Although great efforts (e.g. evolution of computerised planning systems and dose-delivery systems, increasing radiosensitivity with chemotherapy, DNA-targeting agents, antimetabolic agents, and antiangiogenics) have been made to increase the radiotherapeutic effect, the pathological complete response is only 4–30% in most locally advanced cancers [2, 3].

The success of immunotherapy has greatly encouraged researchers to combine it with radiotherapy to improve tumor control. However, the optimal sequence of immunotherapy and radiotherapy remains unclear. Multiple preclinical models have demonstrated that concurrent administration of radiotherapy and immune checkpoint inhibitors (e.g. antibodies targeting PD-1/PD-L1, CTLA-4, and GITR) results in better tumour control and survival benefit [4–6]. These are based on the conventional theory that radiotherapy enhances immunotherapy response through multiple mechanisms (e.g. creating neoantigens and T-cell receptor repertoire, inducing tumour-associated neoantigen presentation, and activating the cGAS-STING pathway) [7, 8]. However, clinical trials have reported conflicting results. In the PEMBRO-RT (phase 2) and MDACC (phase 1/2) trials, adding radiotherapy to pembrolizumab immunotherapy significantly improved outcomes in patients with metastatic non-small-cell lung cancer [9, 10]. Prospective studies incorporating concurrent immune checkpoint inhibitors and chemoradiotherapy in locally advanced head and neck squamous cell cancer and non-small cell lung cancer (PACIFC-2) did not improve outcomes [11, 12].

Radioresistance is a major cause of failure of radiotherapy. Hypoxia, induced by abnormal tumour vessels and impaired perfusion, is an adverse prognostic feature that plays a crucial role in radiotherapy resistance [13–15]. Morphological, genetic, and functional abnormalities in tumour endothelial cells (TECs) contribute to the formation of aberrant tumour vasculature. These vascular irregularities directly induce hypoxic microenvironments, thereby activating the HIF-1α/VEGF signalling pathway. The resultant upregulation of this pathway further exacerbates vascular dysfunction and impairs tissue perfusion, creating a self-reinforcing pathological cycle [16]. Traditional anti-angiogenic therapy can inhibit tumour vessel growth and retard tumour progression depending on the dose and time of drug administration, but paradoxically increases metastasis [17–19]. In addition, current anti-angiogenic therapies (such as VEGF-targeted agents) do not improve overall survival when added to chemoradiation [20, 21]. Therefore, vessel normalization is emerging as a novel anti-tumour paradigm that aims to restore perfusion and reverse hypoxia [22]. In the past decade, an increasing number of researchers have confirmed that host immunity status (e.g. regulatory T cell level, myeloid-derived suppressor cell level, expression of coinhibitory ligands such as PD-L1, chemokine signature) regulates tumour radiosensitivity [23–26]. Recent phase II trials investigating the incorporation of PD-1 inhibitors before radiotherapy have shown promising benefits for locally advanced ESCC and locally advanced stage hypopharyngeal carcinoma [27, 28]. Until now, whether immunotherapy can act as a radiation sensitizer has not been clarified in animal models or clinical trials.

In this study, we showed that anti-PD-1 administration resulted in radiosensitization. The Anti-tumour response was more pronounced after induction of anti-PD-1 therapy combined with concurrent radioimmunotherapy compared with concurrent radioimmunotherapy. Mechanistically, induction of anti-PD-1 therapy rescued intratumor hypoxia through vessel normalization in a CD4^+^ T cell-dependent manner and ultimately sensitized the cells to radiation.

## Materials and methods

### Study design

This research aimed to examine the optimal sequence of anti-PD-1 therapy combined with local tumour radiotherapy to enhance radiosensitivity. All animals were randomly assigned to different treatment groups, and the investigators were blinded to all groups.

### Cell lines and culture

The murine breast cancer cell line 4T1 and the murine Lewis lung cancer cell line LLC were obtained from the Chinese Academy of Sciences Cell Bank. 4T1 and LLC cells were maintained in RPMI 1640 (Invitrogen, Carlsbad, CA, USA) and DMEM (Invitrogen, Carlsbad, CA, USA), respectively, containing 10% fetal bovine serum (Gibco, Auckland, New Zealand) in a humidified incubator at 37 °C with 5% CO_2_. Cell lines were authenticated by Short Tendem Repeat Testing and tested for mycoplasma contamination.

### Animals

This study was approved by the Animal Care and Use Institutional Committee of the Fudan University Shanghai Cancer Center (FUSCC-IACUC-S20210019). All experiments were adhered to ARRIVE guidelines. Female, 6-week-old C57BL/6 or BALB/c mice were purchased from Shanghai JieSiJie Laboratory Animals Co. LTD and housed under SPF conditions. 4T1 or LLC cells were harvested using 0.25% trypsin (HyClone). 1 × 10^6^ LLC cells in 100 μL of normal saline were injected subcutaneously into the right flank of C57BL/6 mice. 4T1 tumour cells (2 × 10^5^) were directly injected into the Balb/c mammary fat pads. Day 0 represents the day of tumour inoculation. Mice assigned to the radiotherapy groups received 10 Gy/5 fractions of irradiation daily for 5 consecutive days (from Day 8 to Day 12) using the Pxi X-RAD320 system [6]. Tumour volume was calculated using the formula *L* × *W* × *W*  × 0.5, where *L* is the longest dimension and *W* is the perpendicular dimension. Tumours were harvested at Day 18. Animals with tumours greater than 20 mm in diameter or no tumours were euthanized. The sample size for each experimental group was chosen based on commonly used sample sizes in similar published studies in our field. All animal experiments were approved by the Animal Ethics Committee of Fudan University Shanghai Cancer Center and were performed in accordance with the institution’s guidelines for the care and use of laboratory animals.

### In vivo treatment

For immune checkpoint blockade therapy, 125 μg anti-PD-1 (clone RMP1-14, #BE0146, BioXCell) or the same amount of isotype-matched control antibody rat IgG2a (clone 2A3, #BE0089, BioXCell) was intraperitoneally injected into the animals. For PD-1 inhibitor group and concurrent treatment group, anti-PD-1 antibodies or isotype were administered on Day 8, Day 10 and Day 12. For induction treatment group, anti-PD-1 antibodies or isotype were administered on Day 4, Day 6 and Day 8. For CD4 T cell or CD8 T cell depletion experiments, anti-PD-1 antibodies or isotype were administered on Day 1 and Day 7.

For antibody depletion experiments, animals received 250 μg of depleting or isotype control monoclonal antibodies, including CD4 (clone YTS177, #BE0003-3, BioXCell), CD8 (clone 53-6.7, #BE0004-1, BioXCell), and rat IgG2a (clone 2A3, #BE0089, BioXCell) to deplete different T cell subsets. Antibodies were administered on Day 3 and Day 1 before tumour inoculation, and then twice weekly.

### Pimonidazole staining

For hypoxia studies, animals received 2 mg pimonidazole (Hypoxyprobe) intravenously, and tumours were harvested after 30 min. For the staining of tumour tissue frozen sections, a Hypoxyprobe Plus Kit was employed [29].

### Immunofluorescence (IF) and multi-marker immunofluorescence (mIF) staining and quantification

The paraffin-embedded tissue sections were deparaffinized by microwave treatment in Tris-EDTA (pH 9.0 IF, and pH 6.0 for mIF) solutions. The sections were immersed in 100 mmol/L NH_4_Cl for 10 min to reduce the background. After being blocked with 10% goat serum, consecutive tissue sections were probed with primary antibodies at 4 °C overnight. Secondary antibodies were then incubated for 1 h at 20 °C. For mIF, CD8 was stained with primary antibody and HRP conjugated Goat anti-rabbit IgG and amplified the signal with FITC-Tyramide. Then, the sections were microwaved in Tris-EDTA (pH 6.0) solution to remove the primary and secondary antibodies. After blocking again, the combined anti-CD4 and anti-CD31 primary antibodies were added and washed, and amplified signal with fluorescent secondary antibodies. Nuclei were stained with DAPI solution (4,6-diamidino-2-phenylindole) for 2 min at 20 °C. The primary antibodies used were anti-HIF-1α (EPR16897, ab179483, Abcam), anti-CD31 (clone MEC13.3, ab256569, Abcam), anti-NG2 (ab5320, Millipore), anti-CD4 (GB13064-2, Servicebio), and anti-CD8 (GB13429, Servicebio). Secondary antibodies included: Alexa-Fluor-488 Goat anti-Rabbit (111-545-003), Alexa-Fluor-594 Goat anti-Rabbit (111-585-003) (both from Jackson Laboratory), HRP conjugated Goat anti-Rabbit (GB23303), Cy3 conjugated Goat Anti-mouse (GB21301), Cy5 conjugated Goat Anti-rabbit (GB27303) (all from Servicebio). FITC-Tyramide (G1222, Servicebio). The stained sections were visualised by a Carl Zeiss Axio Vert.A1 microscope or scanned with Pannoramic MIDI (3DHISTECH). All slides were analysed independently by two pathologists. The positive areas were computed using the ImageJ software. The scanned images were analysed using Indica HALO software (Indica Labs, UK).

### Flow cytometry

Mouse spleen mononuclear cells were separated on a Ficoll gradient (Sigma, USA).

For cell-surface markers, cells were incubated with anti-mouse CD45-PE-Cyanine7 (25-0459-42), CD4-FITC (11-0042-82), CD8-PE-eFluor610 (61-0081-82), or appropriate isotype controls (eBioscience, CA, USA) for 30 min at 4 °C. For intracellular cytokine detection, cells were stimulated with 1× cell stimulation cocktail (eBioscience, CA, USA) for 10–12 h, fixed, and permeabilized using a BD Cytofix/Cytoperm™ Fixation/Permeabilization Solution Kit (eBioscience, CA, USA), and finally stained with IFN-γ-APC or isotype-matched control antibody (eBioscience, CA, USA). Foxp3 transcription factor staining buffer kits (Thermo Fisher, USA) and Foxp3-PE antibody were used to stain Foxp3. The stained cells were analysed using flow cytometry, and data analysis was performed using FlowJo V10.

### RNA sequencing and data analysis

Total RNA was isolated from each tumour sample using an RNAmini kit (Qiagen, Germany). Strand-specific libraries were constructed using the TruSeq RNA sample preparation kit (Illumina, San Diego, CA, USA). RNA sequencing was performed using the Illumina Novaseq 6000 instrument by Genergy Biotechnology Co. Ltd (Shanghai, China).

Raw data were handled by Skewer and data quality was checked using FastQC v0.11.2 (http://www.bioinformatics.babraham.ac.uk/projects/fastqc/). The read length was 2 × 150 bp. Clean reads were aligned to the mouse genome GRCm38.83 using STAR. The expression of the transcript was calculated by FPKM (Fragments Per Kilobase of exon model per million mapped reads). The thresholds for determining differentially expressed genes (DEGs) were *P* < 0.05, and an absolute fold change ≥ 2. DEGs were then chosen for function and signalling pathway enrichment analysis using the GO KEGG and Hallmark database. Pathway analyses (ORA and GSEA) were performed using ClusterProfiler73 R package (v. 4.2.2). Significantly enriched pathways were determined at *P* < 0.05.

The RNA-Seq expression profiles and corresponding information were obtained from TCGA database (https://portal.gdc.cancer.gov/). For paired analysis, human head and neck cancer data were obtained from GSE179730.

### Statistical analysis

All data were analysed using GraphPad prism 9.0.0 and R studio (version 3.6.3), and presented as the mean values ± standard deviation(M ± SD). Differences between the two groups were analysed using a two-tailed Student’s *t*-test. Tumour growth curves were assessed using a two-way ANOVA. Survival-related indicators were assessed using the Kaplan–Meier method. The variance similar between the groups. Statistical significance was set at *P* < 0.05 (two-sided).

## Results

### Induction anti-PD-1 therapy enhanced anti-tumour response of radiotherapy

**Fig. 1:**
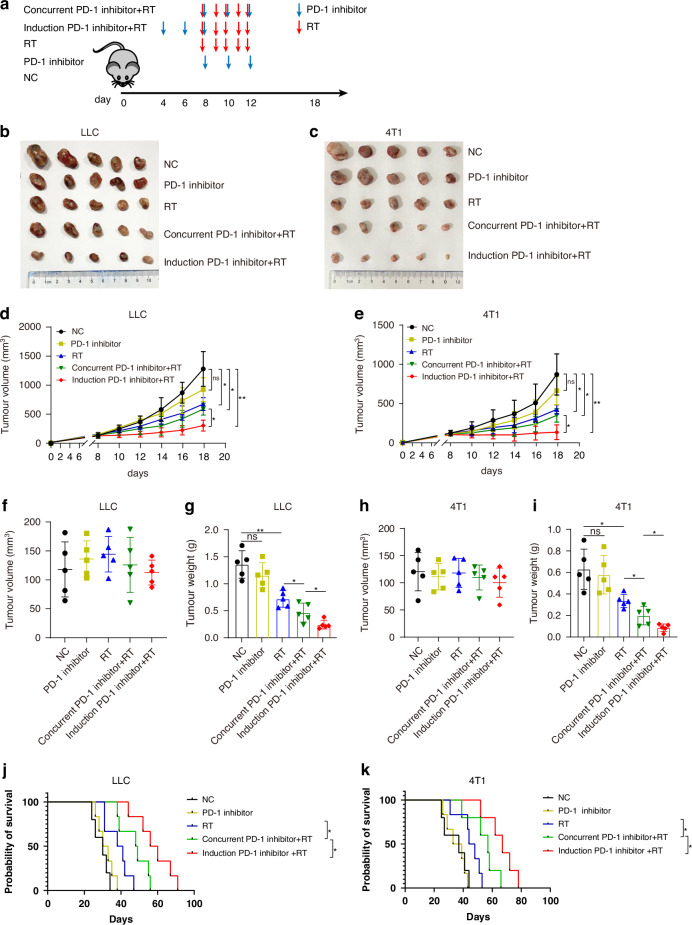
Induction anti-PD-1 therapy combined with radiotherapy maximized anti-tumor response. **a** Treatment schema. **b**, **c** Tumor tissues of Lewis lung cancer (LLC) mouse model and 4T1 breast cancer mouse model were removed at day 18, respectively. **d**, **e** Tumor growth curves of different groups from (**b**, **c**). Data shown are one representative experiment of two independent experiments (*n* = 6 mice per group). Statistical significance was calculated by two-way ANOVA. **f**, **h** The tumor volume before radiotherapy (day 8) of different group in (**b**, **c**), respectively. **g**, **i** The tumor weight of tissues in (**b**, **c**) respectively. **j**, **k** The overall survival curve. Data are presented as means ± SD. *P* values were calculated using two-tailed unpaired Student’s *t*-test (**f**–**i**).

To explore the optimal timing of anti-PD-1 therapy in combination with radiotherapy, we examined the anti-tumor responses to different combination strategies. The treatment schedule is shown in Fig. 1a. There was no significant difference in the tumor volume before radiotherapy between the groups (Fig. 1f, h). Anti-PD-1 therapy showed anti-tumor effects similar to those of the isotype control in both LLC and 4T1 tumor models (Fig. 1b–e). As expected, combined anti-PD-1 therapy and radiotherapy showed stronger anti-tumor effects than anti-PD-1 therapy or radiotherapy alone. Furthermore, compared to concurrent radioimmunotherapy, induction of anti-PD-1 therapy combined with radiotherapy resulted in better tumor control (Fig. 1d, e, g, i) and overall survival (Fig. 1j, k). Therefore, induction of anti-PD-1 therapy enhanced radiosensitivity compared with concurrent immunotherapy.

### Anti-PD-1 therapy increased radiosensitivity through improving hypoxia

**Fig. 2:**
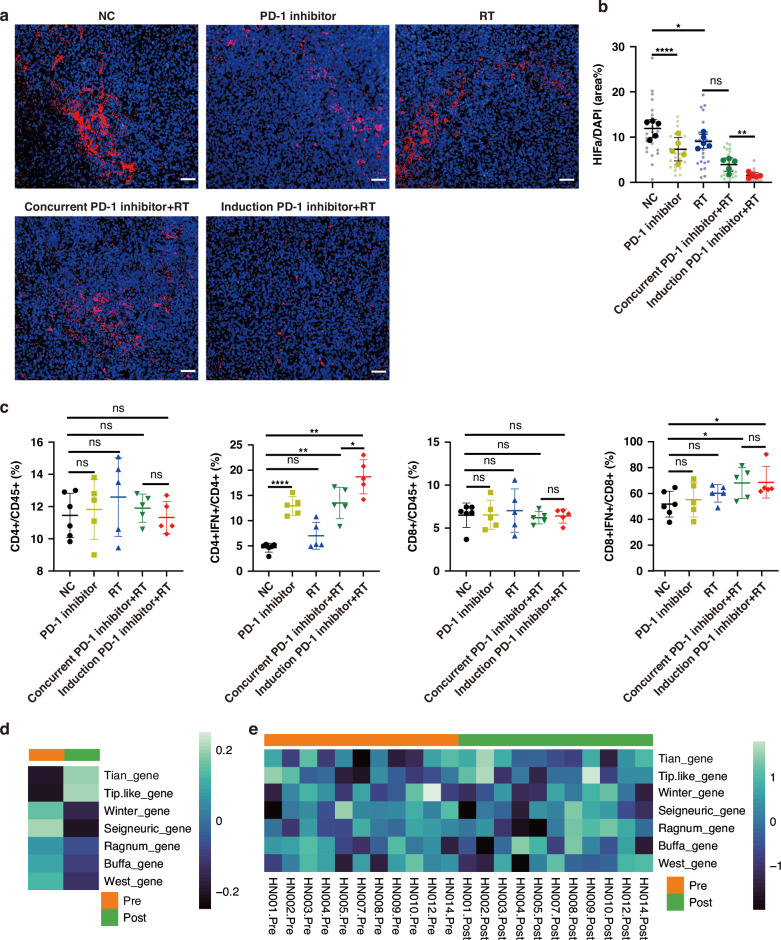
Anti-PD-1 therapy improved hypoxia and CD4^+^ IFNγ^+^ T cells. **a**, **b** Representative images and quantification of tumor hypoxia with HIF-1α (Scale bars, 50 μm). Data are presented as means ± SD. **c** Immune cell profile in spleen. **d**, **e** Hypoxia score, Tian angiogenesis score and Tip-like cells score before and after immunotherapy (*n* = 11 in head and neck cancer). Data are representative of two independent experiments and presented as means ± SD. *P* values were calculated using two-tailed unpaired Student’s *t*-test.

Hypoxia is the hallmark of radioresistance. Our data indicated that anti-PD-1 therapy markedly decreased the hypoxia marker HIF-1α, although the tumor volume was not decreased by anti-PD-1 therapy (Fig. 2a, b). More importantly, hypoxia levels were much lower after induction of anti-PD-1 therapy combined with radiotherapy than after concurrent anti-PD-1 therapy combined with radiotherapy, although the intensities of these two treatments were similar (Fig. 2a, b). Hypoxic tumor microenvironment has been implicated in resistance to immunotherapy. Next, we analysed immune cell profiles after anti-PD-1 treatment. The proportions of CD4^+^ and CD8^+^ T cells were similar among the different treatments. Although both radiotherapy and anti-PD-1 therapy were proven to stimulate the immune response, our data showed that anti-PD-1 therapy, but not radiotherapy, upregulated the proportion of CD4^+^ IFNγ^+^ T cells in the spleen (Fig. 2c, and Supplementary Fig. 1). Induction of anti-PD-1 therapy combined with radiotherapy and concurrent radioimmunotherapy upregulated CD8^+^ IFNγ^+^ T cells and CD4^+^ IFNγ^+^ T cells. Notably, the number of CD4^+^ IFNγ^+^ T cells increased significantly after induction of anti-PD-1 therapy combined with radiotherapy compared to concurrent radioimmunotherapy. Therefore, anti-PD-1 therapy improved hypoxia and radiosensitivity in a CD4+ T-dependent manner PD-1.

Next, we sought to identify the hypoxic characteristics after PD-1 blockade. Hypoxia scores were calculated using mRNA-based signatures as previously described [30]. We analysed hypoxia and angiogenesis using Seigneuric, Buffa, Winter, West, and Ragnum hypoxia signatures, Tian angiogenesis signatures, and Tip-like cell signatures [29, 31–35]. In head and neck cancer (GSE179730), anti-PD-1 (nivolumab) therapy decreased the hypoxia score, whereas the Tian angiogenesis score and Tip-like cell score were upregulated by anti-PD-1 (Fig. 2d, e). These data suggested that anti-PD-1therapy alone regulated tumor-associated hypoxia and angiogenesis.

### CD4^+^ T cells contributed to vessel normalization after anti-PD-1 therapy

**Fig. 3:**
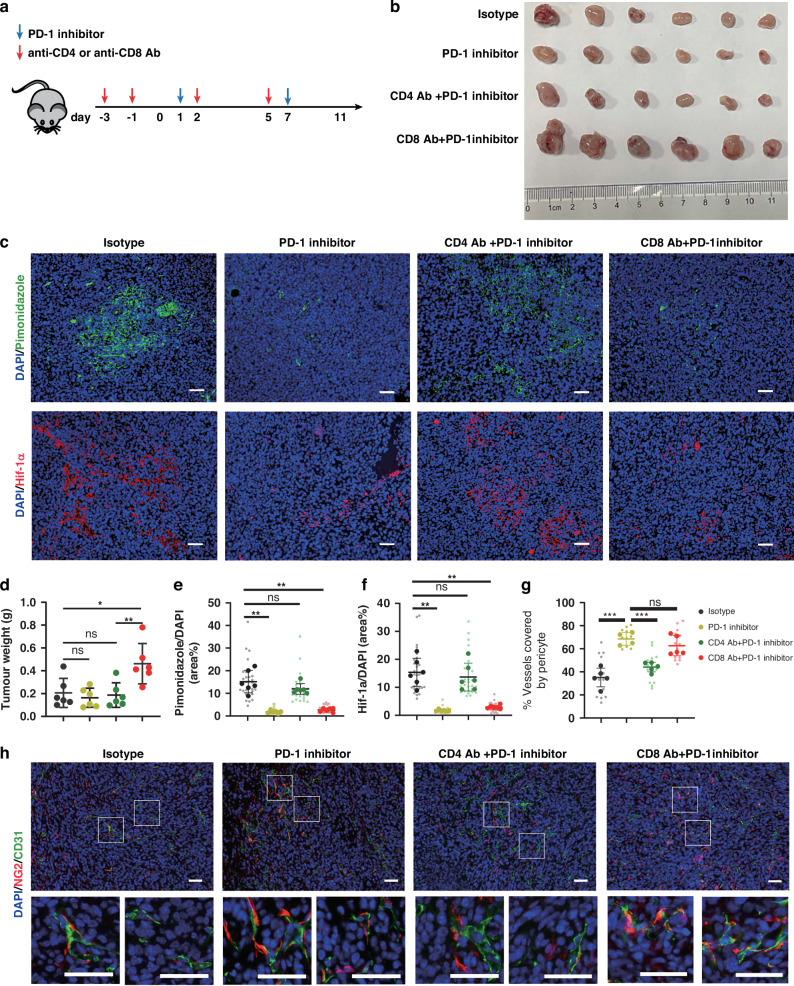
Anti-PD-1 therapy improved hypoxia and promoted vessel normalization. **a** Treatment schema. **b** Tumor tissues of 4T1 breast cancer mouse model removed at day 11. **c** Representative images and quantification of tumor hypoxia with HIF-1α and Pimonidazole (Scale bars, 50 μm). **d** Analyses of tumor weight in (**b**) (*n* = 6 mice per group). **e**, **f** Analyses of tumor hypoxia in (**c**) (n = 6 mice per group). **g**, **h** Immunofluorescence quantification of percentage endothelial cells (CD31^+^) attached by pericytes (NG2^+^) (*n* = 4 to 5 mice per group). Data were representative of two independent experiments and presented as means ± SD. *P* values were calculated using two-tailed unpaired Student’s *t*-test (**d**–**g**).

Vessel normalization is a favourable event during angiogenesis, with improved perfusion and oxygenation. To further validate the effect of anti-PD-1 therapy on hypoxia and vessel normalization during radiotherapy sensitization, animals were treated with PD-1 antibody or isotype control (Fig. 3a). CD31^+^ tumor-associated endothelial cells and NG2^+^ pericytes were profiled. PD-1 therapy decreased hypoxia (HIF-1α and pimonidazole) (Fig. 3c, e, f) and increased the pericyte-endothelial cell ratio (Fig. 3h, g). To study whether intratumor T cells are involved in the regulation of radiosensitivity through vessel normalization after anti-PD-1 therapy, we utilised CD4^+^ or CD8^+^ T cell-depleted animals (Fig. 3a) [29]. After anti-PD-1 treatment, the tumor burden was much higher in CD8^+^ T cell-depleted animals, while CD4^+^ T cell depletion showed no difference with PD-1 blockade (Fig. 3b, d). CD4^+^ T cell depletion blunted the effect of anti-PD-1therapy-induced vessel normalization, with increased hypoxia (HIF-1α and pimonidazole) (Fig. 3c, e, f) and lower pericyte: TEC ratio (Fig. 3h, g). However, vessel normalization in CD8^+^ T cell depletion animals after anti-PD-1 treatment was similar to that of PD-1 blockade.

**Fig. 4:**
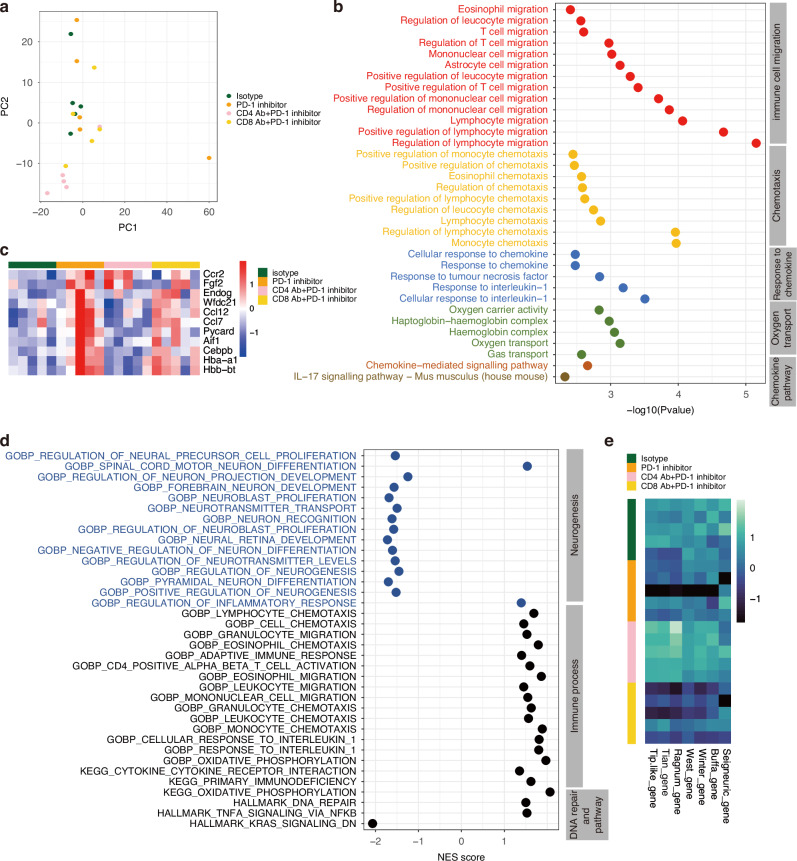
Anti-PD-1 therapy alter transcriptomes. **a** PCA of transcriptional profiles in tumor samples treated with or without anti-PD-1 inhibitors, calculated based on top 1% most variable genes. **b** Dotplot representation of selected significant pathways from GOBP databases on DEGs in isotype treated vs. anti-PD-1 inhibitor treated tumors (ORA, cut-off: *P*. adj<0.05). **c** Heatmap of selected DEGs in isotype treated vs. anti-PD-1 inhibitor treated tumors. **d** Dotplot representation of selected significant pathways from GSEA vs. Hallmark and GOBP databases. GSEA computed by comparing isotype treated vs. anti-PD-1 inhibitor treated tumors (cut-off: *P*. adj<0.05). **e** Heatmap of hypoxia scores (Seigneuric gene, Buffa gene, Winter gene, West gene, Ragnum gene), Tian anagenesis scores and Tip-like cell scores in tumor samples treated with or without anti-PD-1 inhibitors.

We also performed RNA-seq of mouse tumours after anti-PD-1 treatment. Principal component analysis (PCA) showed that CD4-depletion tumours had very distinct transcriptional profiles compared with non-CD4-depletion tumours (Fig. 4a). However, the transcriptional profiles overlapped between PD-1 inhibitor-treated tumours and isotype-treated tumours, which may be because PD-1 inhibitors mainly reprogram immune cells, and immune cells are not the majority cell population in tumours. The DEGs with significant differences and |Fold Change| > 2 between the isotype control and PD-1 inhibitor groups were selected for functional analysis. PD-1 inhibitor-treated tumours showed significant enrichment in pathways associated with immune cell migration, chemotaxis, response to chemokines, oxygen transport, and IL-17 signalling, suggesting a regulation of oxygen homoeostasis and oxygen immune coupling (Fig. 4b–d). Additionally, PD-1 inhibitors upregulated DNA repair pathways that are related to radiosensitivity. When analysing hypoxia and angiogenesis using Seigneuric, Buffa, Winter, West, and Ragnum hypoxia signatures, Tian angiogenesis signatures, and Tip-like cell signatures [29, 31–35], the PD-1 inhibitor downregulated the hypoxia score, angiogenesis score, and Tip-like cell score, while these functions of PD-1 inhibitor were weakened upon CD4^+^ T depletion (Fig. 4e). These data demonstrated that anti-PD-1 therapy sensitized radiotherapy through vessel normalization in a CD4^+^ T cell-dependent manner.

### Spatial distribution of CD4^+^ T cells after anti-PD-1 therapy

**Fig. 5:**
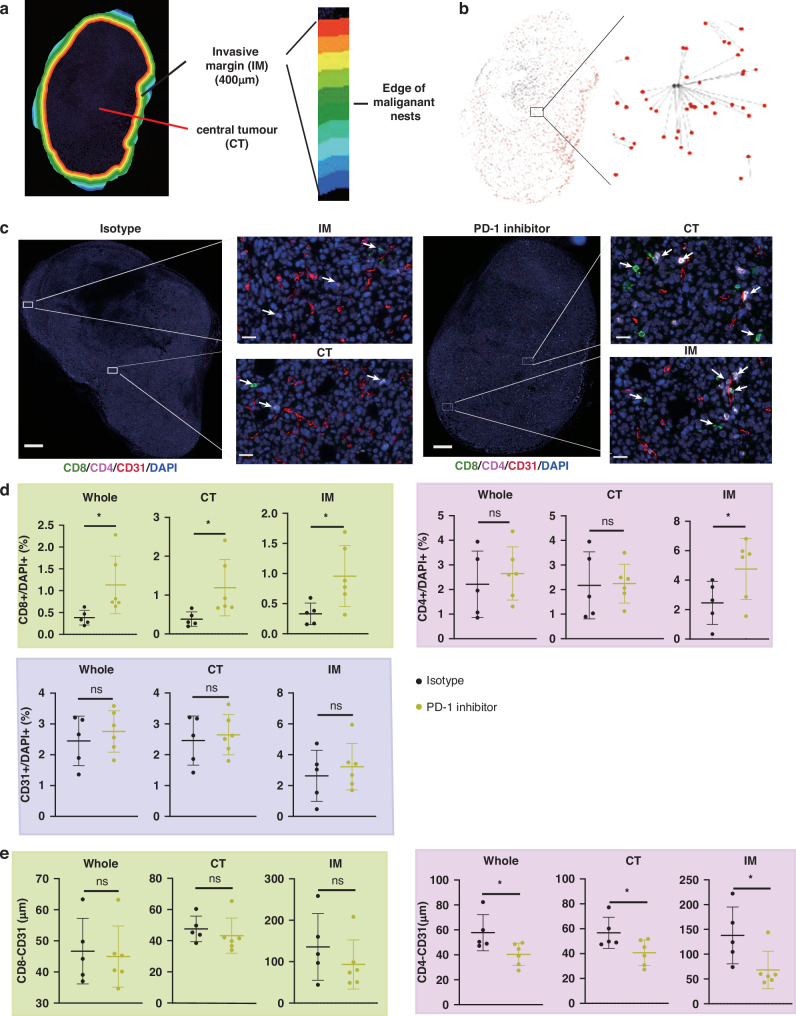
Spatial relationships between tumor infiltration T cells and CD31^+^ endothelial cells. **a** Diagram of central tumor (CT) and invasive margin (IM). The color region represented IM, which was the edge of malignant nests ±200 μm. **b** Diagram of distance analysis by HALO. Grey dots are CD31^+^ cells and red dots are CD4^+^ cells. This was related to (**e**). **c** Scanning images of whole tumor sections (*n* = 5 mice per group). Representative areas are magnified. The bar is 500 μm in left whole tumor images and 20 μm in right magnified images. **d** Analyses of CD8^+^/DAPI^+^, CD4^+^/DAPI^+^, CD31^+^/DAPI^+^ in CT and IM. **e** Analyses of the distance of CD4^+^ T cells or CD8^+^ T cells to the nearest CD31^+^ endothelial cells. *P* values were calculated using two-tailed unpaired Student’s *t*-test (**d**, **e**).

Given that spatial immunophenotypes predict tumor outcomes and immunotherapy responses [36, 37], we sought to determine the relative distribution characteristics of CD4^+^ T cells and vessels after anti-PD-1 therapy. The in situ adaptive immune response in the centre of the tumor (CT) and invasive margin (IM) and the distance between the two cells were analysed by HALO (Fig. 5a, b). Immunostaining for CD4^+^ T cells, CD8^+^ T cells, and CD31^+^ endothelial cells was quantified using a HALO workstation. Consistent with previous studies, PD-1 inhibitors increased tumor infiltration of CD8^+^ T cells in both the CT and IM areas (Fig. 5c, d). We only observed an increase in CD4^+^ T cells in the IM area, suggesting a unique infiltration pattern of CD4^+^ T cells (Fig. 5c, d). The density of CD31^+^ endothelial cells was not regulated by PD-1 inhibitor, suggesting PD-1 induced vessel normalization was not related to vascular pruning (Fig. 5c, d). The relative spatial positions of CD8^+^ T cells and CD31^+^ endothelial cells were similar between the isotype and PD-1 inhibitor groups. After anti-PD-1 therapy, CD4^+^ T cells were more frequent around CD31^+^ endothelial cells in both CT and IM, and partly co-localized with CD31^+^ vessels, suggesting direct crosstalk between CD4^+^ T cells and CD31^+^ endothelial cells (Fig. 5c, e).

## Discussion

Recent success of immunotherapy for metastatic solid cancers has resulted in trials exploring the efficacy to prevent or delay relapse following combination therapy of immunotherapy and radiotherapy in locally advanced diseases. Most studies have focused on how radiation acts as an immunotherapy sensitizer, but whether immunotherapy can enhance the therapeutic effects of radiation has not been widely investigated. This study demonstrated that the efficacy of radiotherapy can be improved by induction immunotherapy in preclinical models. We further analysed the gene expression profiles in human and mouse tumor datasets and found that immunotherapy decreased hypoxia and promoted vessel normalization, which was verified by an in vivo study. Through the application of anti-CD4 or anti-CD8 neutralizing antibody, we proved that CD4^+^ T cells contributed to the vessel normalization and radiosensitization function of induction PD-1blockade. Strikingly, immunotherapy-related spatial immunovascular phenotype confirmed the vital role of CD4^+^ T cells in improvement of the tumor hypoxia.

Recently, several studies have shown that immunotherapy alone has a limited effect on tumor suppression, but a combination of immunotherapy and radiotherapy delays tumor progression more effectively than either treatment alone, similar to our data [6, 38, 39]. Preclinical and clinical studies have suggested that immunotherapy may be most effective when administered earlier. In animal models, the early application of immunotherapy showed greater therapeutic efficacy during concurrent radioimmunotherapy and tumor resection [6, 40, 41]. The PACIFIC study indicated that early application of immunotherapy improved patient survival in an adjuvant setting [42]. Strikingly, our data expanded on these findings by evaluating the anti-tumor response of induction immunotherapy combined with radiotherapy, and proved that PD-1 blockade before radiation pronounced the radiation effect. Although a number of clinical studies have assessed the efficacy of induction immunotherapy, the aim was to evaluate the efficacy of induction immunotherapy combined with radiotherapy or chemotherapy, and only one trial (NCT05267392) explored the efficacy of an anti-PD-L1 (durvalumab) agent as induction therapy followed by radical radiotherapy or chemoradiotherapy. Therefore, this study has inspired researchers to set up clinical trials to determine whether induction immunotherapy could further improve survival outcomes over concurrent immunotherapy.

In this study, we demonstrated that anti-PD-1 treatment increased pericyte coverage and improved hypoxia alleviation, but did not significantly modulate endothelial density. Consistent with these findings, perfusion functional imaging in human esophageal cancer revealed improved blood perfusion without excessive vascular pruning following immunotherapy [28]. In contrast, anti-VEGF therapy promotes vascular normalization primarily through VEGF signalling-mediated vessel pruning, as indicated by reduced endothelial density. Conversely, anti-PD-1-induced vascular normalization appears to be mediated by T helper cells [29]. Thus, while infiltration of specific immune cells following anti-PD-1 treatment may enhance intratumoral perfusion and pericyte coverage, it does not directly engage the VEGF pathway and therefore may not elicit vascular pruning.

Subsequently, we characterised the correlation between radiosensitivity and PD-1 blockade. Hypoxia is a hallmark of radioresistance and mainly results from abnormal tumor vessels [3, 43]. The conventional concept states that vessel normalization could resolve hypoxia and enhance immune cell infiltration, increasing the efficacy of immunotherapy [44, 45]. On the other hand, immune microenvironment contributed to angiogenesis via suppressive cells including regulatory T cells, myeloid-derived suppressor cells, or M2-tumor-associated macrophages [46]. Given that immunotherapy reactivates the immune system, it is reasonable to suppose that it may promote vessel normalization by remodeling the tumor immune microenvironment. As expected, a single dose of neoadjuvant PD-1 blockade induced high expression of genes from Gene Ontology involved in T cell activation and angiogenesis pathways [47]. PD-1 blockade regulates tumor vessel normalization and eradicates metastasis through CD4^+^ T cells [29]. In addition, depletion of CD4^+^ T cells negated the efficacy of neoadjuvant immunotherapy in an animal study [40]. Here, we uncovered the potential mechanisms by which anti-PD-1 therapy promoted vessel normalization, decreased hypoxia in a CD4^+^ cell-dependent manner, and further enhanced radiosensitivity. A previous study confirmed that anti-PD-1 monotherapy enhanced tumor cell killing by restimulating effector CD8^+^ T cells [48–50]. The results of our current study expand the function of immunotherapy to enhance radiosensitivity.

Finally, we performed spatial analysis and illustrated unique CD4^+^ T cell and CD31^+^ endothelial cell ‘pairs’ induced by anti-PD-1 therapy. Clinical studies have also shown that the spatial distribution patterns of immune cells are associated with clinical outcomes and immunotherapy responses [36, 51]. Furthermore, immune cells display different functions in different regions, and those in hypoxic regions regulate vessel normalization [52]. Here, we describe that the CD4^+^ T cells and CD31^+^ endothelia cells ‘pairs’ is a signature of anti-PD-1immunotherapy, but the molecular mechanism of interaction between CD4^+^ T cells and CD31^+^ endothelia cells still need to explore. To date, most researchers have performed spatial studies according to the anatomical structure of the tumor, and zoning design according to the function signature may improve the tumor microenvironment.

The combination of immunotherapy and radiotherapy is a promising strategy and is well tolerated; however, the optimal treatment schedule has not yet been clearly defined. Our study emphasizes the importance of optimizing the timing of immunotherapy during immunoradiotherapy, and administration of immunotherapy in the induction setting remains to be further elucidated in clinical trials. In future clinical trials comparing induction and concurrent immunotherapy, it will be important to identify and measure intratumor T cells and vessel normalization to determine whether they can act as biomarkers to distinguish patients who will derive long-term benefits. Positive trial results will further revolutionize the field of cancer immunotherapy and improve outcomes in patients with cancer.

## Supplementary information

supplementary figure legends

Supplement figure 1

Supplement figure 2

## Data Availability

The data are available from the corresponding author (K.L.Z) on reasonable request.
