# Supplementary material for: PD-1 inhibitor improves radiosensitivity by tumor vessel normalization
Source: Br J Cancer. 2025 Dec 26;134(5):820–30. doi: 10.1038/s41416-025-03315-8 (PMC12905340; doi:10.1038/s41416-025-03315-8)
Supplement: Supplementary file 1 — supplementary figure legends [file 41416_2025_3315_MOESM1_ESM.docx]

Supplementary figure legends

Supplementary figure 1. Gating strategy. Gating strategy for figure 2C.

Supplementary figure 2. Body weight curves of different groups. Body weight changes in different groups of LLC (Figure 1D) and 4T1 (Figure 1E) tumor models.
