# Supplementary figures and images for: PD-1 inhibitor improves radiosensitivity by tumor vessel normalization
Source: Br J Cancer. 2025 Dec 26;134(5):820–30. doi: 10.1038/s41416-025-03315-8 (PMC12905340; doi:10.1038/s41416-025-03315-8)

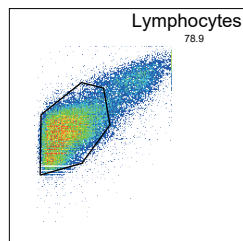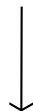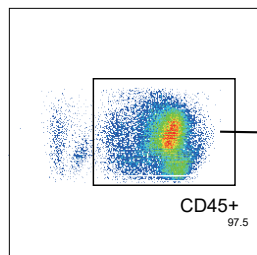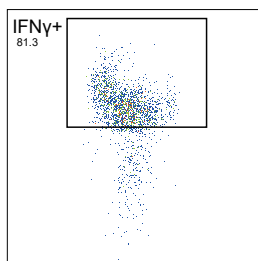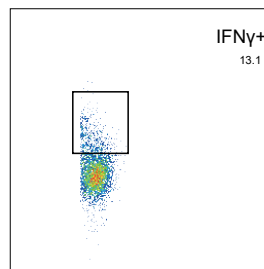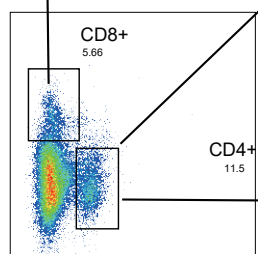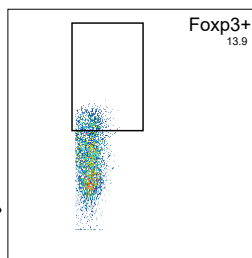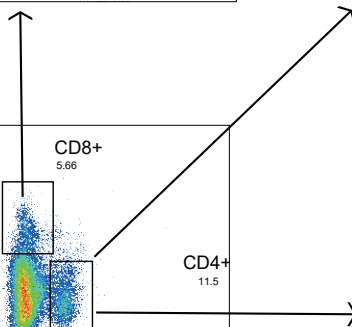

Supplement: Supplementary file 2 — Supplement figure 1 [file 41416_2025_3315_MOESM2_ESM.pdf]

## LLC

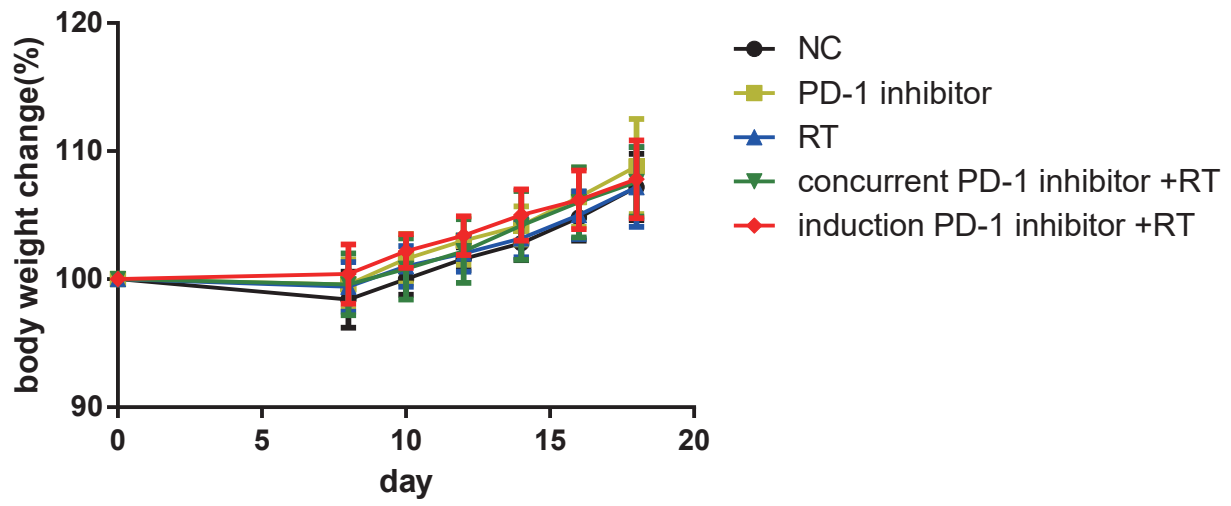

## 4T1

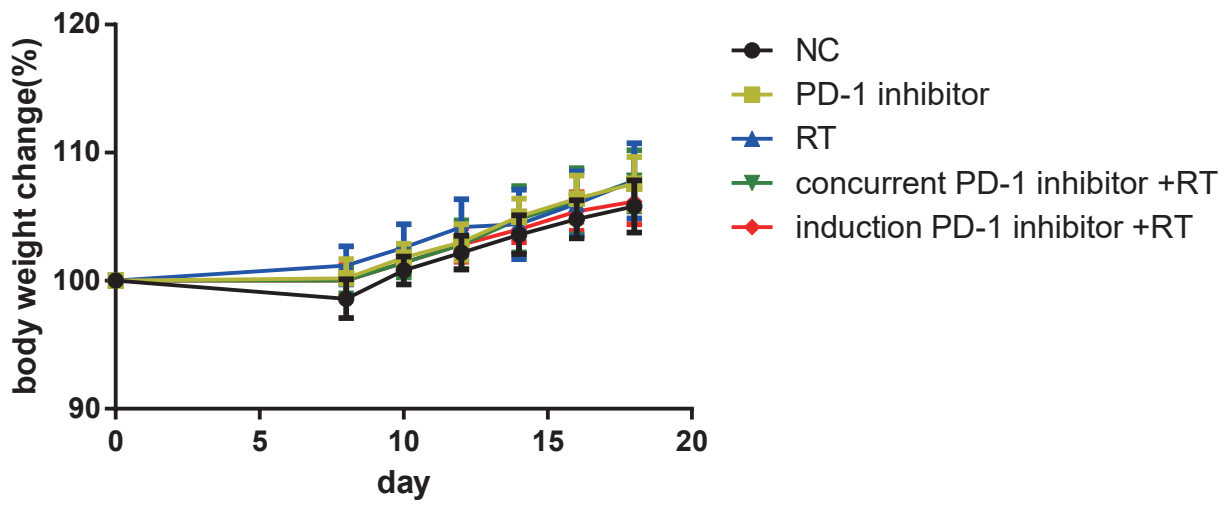

Supplement: Supplementary file 3 — Supplement figure 2 [file 41416_2025_3315_MOESM3_ESM.pdf]
